# Tuberculous ciliary body granuloma initially diagnosed as bullous retinal detachment: a case report

**DOI:** 10.1186/s12886-024-03503-9

**Published:** 2024-06-06

**Authors:** Jie Zhu, Huirong Xu, Qing Chang, Fang Chen

**Affiliations:** 1 Department of Ophthalmology, Northern Jiangsu People’s Hospital, Yangzhou, Jiangsu Province China; 2 Department of Ophthalmology, The Eighth Affiliated Hospital of Sun Yat-Sen University, Shenzhen, Guangdong Province China; 3 Department of Ophthalmology, Fudan University Eye and ENT Hospital, Fudan University, Shanghai, China

**Keywords:** Choroiditis, Ocular tuberculosis, Retinal detachment, Tuberculosis, Uveitis, Ciliary body granuloma

## Abstract

**Background:**

Ocular tuberculosis is a relatively rare extrapulmonary manifestation of tuberculosis. This vision-threatening disease is extremely challenging to diagnose, particularly because it can mimic other diseases. We report a case of tuberculous ciliary body granuloma initially diagnosed as bullous retinal detachment.

**Case report:**

A 52-year-old female presented with bullous retinal detachment in her left eye, and ultrasound biomicroscopy (UBM) verified the presence of a lesion with ciliary body granulomatous inflammation. The T-SPOT was positive, and the purified protein derivative (PPD) test was strongly positive (diameter of 20 mm). Following the administration of oral anti-tuberculosis regimen combined with prednisone, the retina gradually became reattached, the ciliary body granuloma became significantly reduced in size, and the visual acuity of the patient noticeably improved.

**Conclusions:**

Tuberculous ciliary body granulomas can cause bullous exudative retinal detachment and can be diagnosed with UBM. Early and full-course anti-tuberculosis treatment (ATT) combined with corticosteroid therapy can improve the patient prognosis.

## Background

Ocular tuberculosis is a rare presentation of extrapulmonary infection caused by *Mycobacterium tuberculosis (M. tuberculosis. MTB)*. Due to the diversity of clinical presentations and the low positive rate of etiological diagnosis, it can be very difficult and challenging to diagnose this disease. Ciliary body granulomas are particularly rare in patients with ocular tuberculosis. We report a case of tuberculous ciliary body granuloma initially diagnosed as bullous retinal detachment (RD).

## Case presentation

**Fig. 1:**
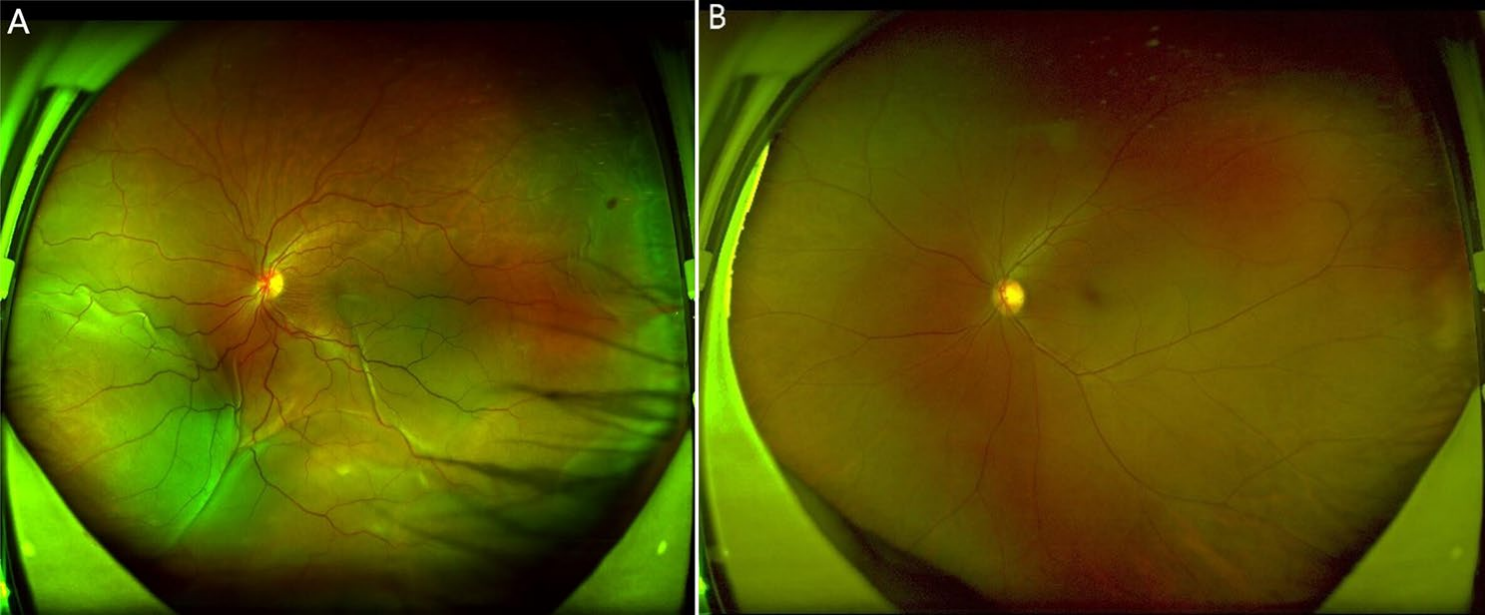
Color fundus photographs of OS at the time of initial diagnosis revealed bullous retinal detachment and no retinal breaks were observed (1**A**). Seven months after treatment, the retina was completely reattached (1**B**)

**Fig. 2:**
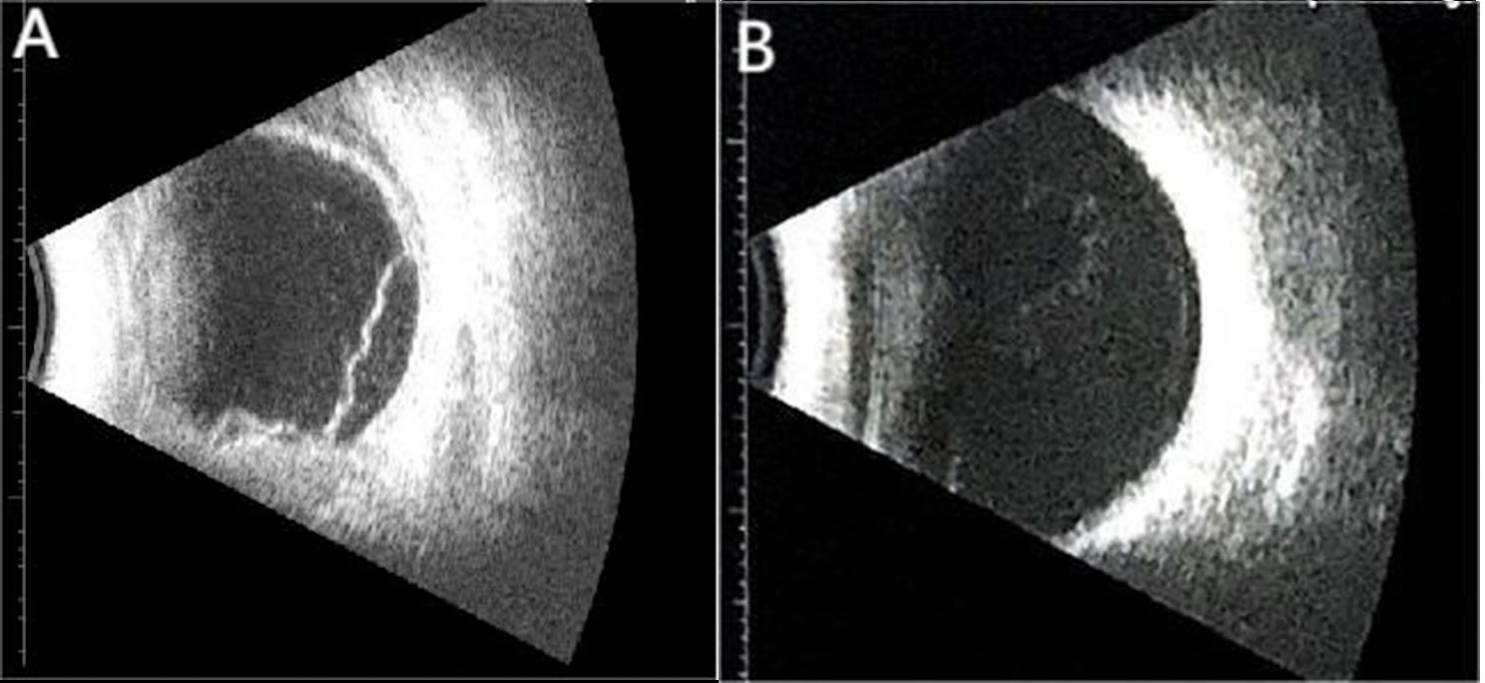
Ultrasonography(B-scan) at the time of initial diagnosis showed thickening of the sclera and hyper-echoic banding in the temporal and inferior quadrants, consistent with retinal detachment(2**A**). Eight months after stopping treatment, the retina was completely reattached (2**B**)

**Fig. 3:**
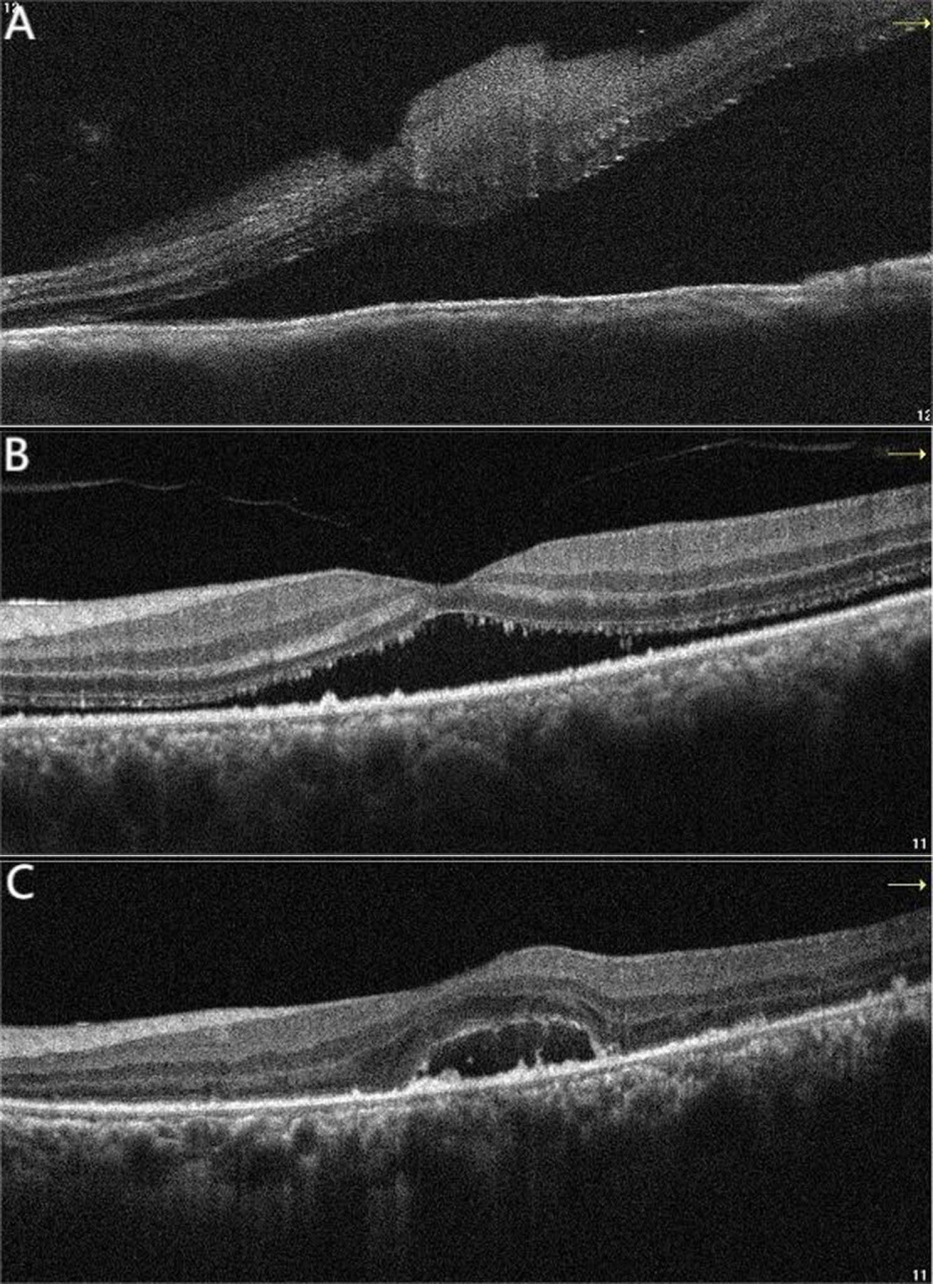
OCT at the time of initial diagnosis revealed subretinal fluid in the macula OS (3**A**). One month later, OCT revealed significant resolution in the subretinal fluid (3**B**). Seven months after treatment, OCT showed minimal residual fluid in the fovea. (3**C**)

**Fig. 4:**
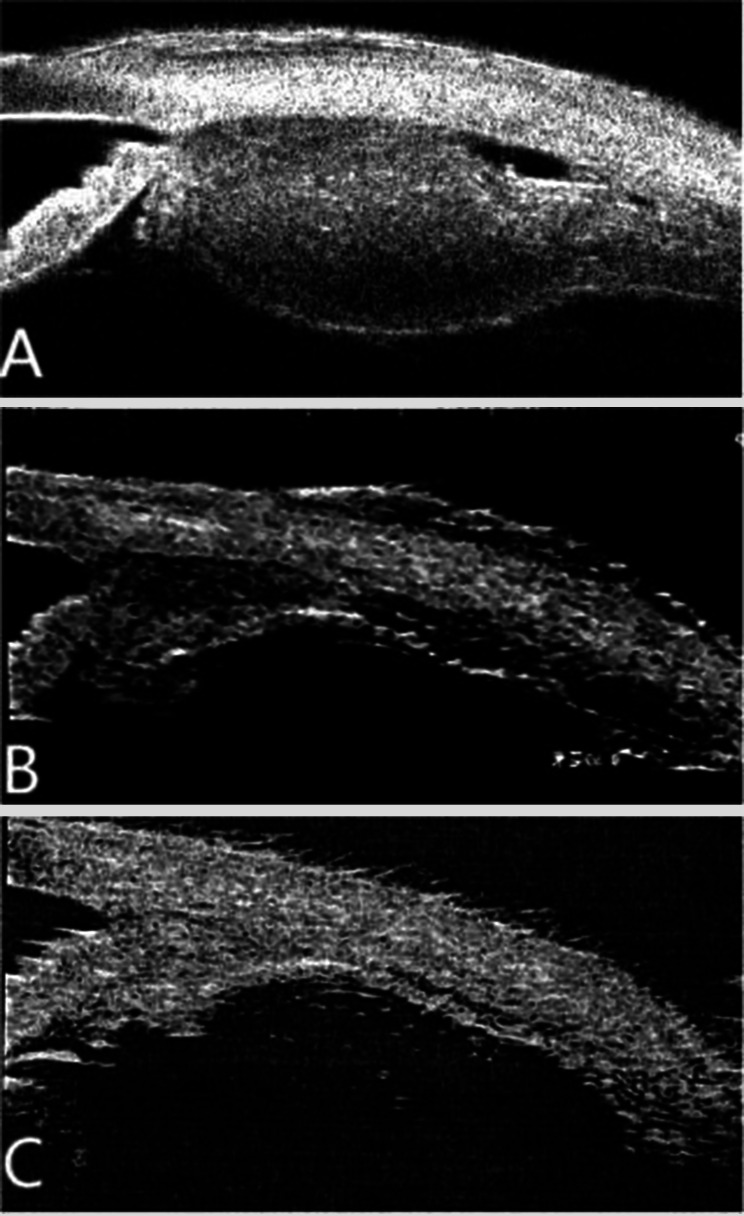
UBM at the time of initial diagnosis revealed a lesion with ciliary body granulomatous inflammation measuring 9.25*1.67*12.92 mm (4**A**). Seven months after treatment, UBM showed a significant reduction in the size of the ciliary body granuloma to approximately 7.82*0.75*2.54 mm (4**B**). Eight months after stopping treatment, UBM suggested that the size of the ciliary body granuloma was 1.96*0.23*1.16 mm (4**C**)

A 52-year-old female patient with a chief complaint of visual disturbance in her left eye (OS) for four days was referred to our ophthalmology department. She denied a past relevant medical history. Ocular examination revealed that the best corrected visual acuity (BCVA) of the right eye (OD) was 20/20, while that of the left eye was 20/100. The intraocular pressure in both eyes was normal, and the right eye was unremarkable. Slit-lamp biomicroscopy revealed conjunctival hyperaemia and 2 + anterior chamber cells with 2 + flare OS. The vitreous had 2 + cells and 2 + haze OS. Fundus examination revealed bullous RD with subretinal fluid, which shifted with changes in body position. No retinal breaks were observed (Fig. 1A). Ultrasonography (B-scan) revealed thickening of the sclera and hyper-echoic banding in the temporal and inferior quadrants, consistent with retinal detachment (Fig. 2A). Optical coherence tomography (OCT) revealed subretinal fluid in the macula OS (Fig. 3A). The anterior chamber depth was 2.25 mm. Ultrasound biomicroscopy (UBM) demonstrated diffuse thickening of the ciliary body and choroid, with a mass of low echogenicity in the pars plana and pars plicate; the internal structure was inhomogeneous, and the boundary was unclear. A lesion with ciliary body granulomatous inflammation measuring 9.25*1.67*12.92 mm was confirmed (Fig. 4A). Fundus fluorescein angiography (FFA) revealed non-perfusion zones with accumulated fluorescence. The results of indocyanine green angiography (ICGA) were unremarkable. We carried out comprehensive systemic examinations, including serological tests for infectious and rheumatological disorders such as hepatitis, syphilis and tuberculosis. All results were negative except for the results of T-cell-based release assay (T-Spot®.TB, T-SPOT) test, which were positive, and those of the purified protein derivative (PPD) test, which were strongly positive (with an induration diameter of 20 mm). Chest computed tomography (CT) revealed multiple pulmonary nodules, some of which were ground-glass opacities. A consultation from the Infectious Diseases Department was sought to evaluate the patient and ruled out active tuberculosis, as the patient had no systemic symptoms.

According to the patient’s medical history and clinical findings, we considered a diagnosis of tuberculous ciliary body granuloma (clinically suspected). Anti-tuberculosis regimens, including isoniazid, rifampicin, ethambutol, and pyrazinamide combined with prednisone (oral 40 mg/d, tapering according to the patient’s condition), were prescribed. One month later, the BCVA OS was 20/100, and the anterior chamber cells and flare were significantly reduced. OCT revealed significant resolution in the subretinal fluid (Fig. 3B). Seven months after initial treatment, the BCVA OS was 20/60, and the retina was completely reattached (Fig. 1B). OCT revealed minimal residual fluid in the fovea (Fig. 3C). UBM revealed a significant reduction in the size of the ciliary body granuloma to approximately 7.82*0.75*2.54 mm (Fig. 4B). The patient stopped anti-tuberculosis treatment (ATT) following one year of consistent therapy. After another eight months, the BCVA was 20/100 OS, and the lens was opaque (Fig. 2B). UBM suggested that the size of the ciliary body granuloma was 1.96*0.23*1.16 mm (Fig. 4C). The BCVA of her left eye reached 20/50 after cataract surgery.

## Discussion

We report an unusual case of ocular tuberculosis, which was initially diagnosed with bullous retinal detachment and finally was identified as a manifestation of tuberculous ciliary body granuloma. There are many clinical manifestations of ocular tuberculosis, such as choroiditis, retinal vasculitis, endophthalmitis, intermediate uveitis, and even isolated macular oedema [1, 2]. Few reports about ocular tuberculosis complicated with retinal detachment have been published. Song [3] reviewed 32 cases of tuberculosis-related retinal detachment and reported that the most common fundus finding associated with exudative RD was choroidal tuberculoma, accounting for 53.1% (17/32) of cases, followed by optic disc oedema and retinal exudation. Exudative RD attributed to tuberculous subretinal abscesses has also been reported recently [4, 5]. Our patient presented with a tuberculous ciliary body granuloma, a rare manifestation of ocular tuberculosis. To date, only a limited number of cases of ciliary body granuloma have been reported [6–9]. To our knowledge, there have been no reported cases of retinal detachment caused by a ciliary body granuloma. According to the consensus statements for initiating ATT for ocular tuberculosis recommended by the Collaborative Ocular Tuberculosis Study (COTS-1) [10], patients who present with tuberculous choroidal granuloma may be considered for ATT based on positive PPD tests or interferon-gamma release assays (IGRAs). The results of both the PPD test and IGRA were positive in this patient. ATT and corticosteroid therapy were prescribed immediately after a clinically suspected diagnosis of tuberculous ciliary body granuloma. The patient’s retina rapidly became reattached, and the ciliary body granuloma became significantly reduced in size. Furthermore, no recurrence was observed after anti-tuberculosis drug withdrawal.

The diagnosis of tuberculous ciliary body granulomas is challenging; the gold standard is histopathological examination through biopsy, but specimens can be difficult to obtain in clinical practice. Therefore, in practical terms, the diagnosis predominantly relies on medical history, multimodal imaging, and diagnostic ATT. UBM is an important imaging tool for auxiliary diagnosis, and it can also help distinguish tuberculous ciliary body granulomas from other ciliary body tumours. The typical UBM manifestations of ciliary body melanocytoma are hyperechogenic masses with characteristic posterior shadows. While ciliary body melanomas usually manifest as regular dome-shaped masses, UBM reveals a medium-low echogenic mass with acoustic attenuation and a homogeneous internal structure [11]. Melanomas may present large cavitations located at the top of the tumour and have invasive features [12]. The UBM findings of tuberculous ciliary body granulomas are different. Elboukhani [13] reported a case of tuberculous ciliary body granuloma, in which UBM showed that the mass had the appearance of a ciliary melanoma with an inhomogeneous internal echo. The mass exhibited a significant reduction in size after ATT. Latiff [8] reported a case of iridociliary tuberculoma in which UBM revealed a heterogeneous iridociliary mass with echolucent spaces within the same quadrant. Singh [6] reported a case of disseminated tuberculosis with concurrent ciliary body granuloma and a thyroid mass. The UBM showed homogenous echogenicity of the ciliary body mass. Our patient’s UBM revealed a hypoechoic, inhomogeneous mass in the pars plana of the ciliary body. A larger cohort of patients is needed to better understand the UBM characteristics of tuberculous ciliary body granulomas.

Due to the limited number of documented cases of ocular tuberculosis presenting with RD and the lack of clarity regarding the underlying mechanisms, a standardized treatment protocol remains to be formulated. Among the 32 eyes with tuberculosis-related retinal detachment reviewed by Song [3], 14 were treated with ATT combined with systemic corticosteroid treatment, only one of which (7.1%) had a decrease in BCVA, while four (40%) of the 10 eyes treated with ATT alone had a decrease in BCVA. The authors believe that, especially for tuberculosis patients with bullous retinal detachment, ATT combined with systemic corticosteroid therapy will yield better visual outcomes than ATT alone or systemic steroids followed by ATT. Some scholars believe that the emergence of exudative RD can indicate that the patient has a pronounced immune response to *M. tuberculosis*. Therefore, systemic corticosteroid therapy should be used simultaneously to reduce inflammation, shorten the duration of exudative RD, and improve patient prognosis [14]. Our patient started on a combination of ATT and corticosteroid therapy at the same time and had a good prognosis.

Tuberculosis-related exudative RD is easily misdiagnosed and improperly treated. Unnecessary surgical interventions may worsen this condition. Among the 32 eyes with tuberculosis-related retinal detachment reported by Song [3], three were treated surgically, including surgery involving subretinal fluid drained via inferotemporal sclerotomy and pars plana vitrectomy. Although all three patients received ATT combined with corticosteroid therapy after the operation, the prognosis was very poor. This suggests that inappropriate surgical interventions may exacerbate ocular tuberculosis, and intraoperative destruction of the blood-retinal barrier may aggravate the intraocular immune response to *M. tuberculosis*. Therefore, for patients with exudative RD of undetermined cause, the possibility of tuberculosis infection must be considered, and surgical interventions should be performed with caution.

## Conclusions

This report describes a clinical case of bullous exudative RD caused by a tuberculous ciliary body granuloma. Although the condition is rare, early and regular ATT combined with corticosteroid therapy could lead to a good prognosis. The case findings highlight the need for ophthalmologists to be aware of the different clinical manifestations of tuberculous ciliary body granulomas. UBM is helpful in diagnosis, which can be verified with the use of effective diagnostic anti-tuberculosis agents.

## Data Availability

All data supporting our findings is contained within the manuscript.

## Abbreviations

UBM: Ultrasound Biomicroscopy
PPD: Purified protein derivative
BCVA: Best-Corrected Visual Acuity
ATT: Anti-Tuberculosis Treatment
RD: Retinal Detachment
OCT: Optical Coherence Tomography
FFA: Fundus Fluorescein Angiography
ICGA: Indocyanine Green Angiography
CT: Computed Tomography
COTS: Collaborative Ocular Tuberculosis Study
PCR: Polymerase Chain Reaction
