# Correction: Tuberculous ciliary body granuloma initially diagnosed as bullous retinal detachment: a case report

**DOI:** 10.1186/s12886-024-03525-3

**Published:** 2024-06-17

**Authors:** Jie Zhu, Huirong Xu, Qing Chang, Fang Chen

**Affiliations:** 1 Department of Ophthalmology, Northern Jiangsu People’s Hospital, Yangzhou, Jiangsu Province China; 2 Department of Ophthalmology, The Eighth Affiliated Hospital of Sun Yat-Sen University, Shenzhen, Guangdong Province China; 3 Department of Ophthalmology, Fudan University Eye and ENT Hospital, Fudan University, Shanghai, China

**Correction: BMC Ophthalmol 24, 236 (2024)**

10.1186/s12886-024-03503-9

In this article [1], due to a typesetting error, the panels in the legends of Figs. 2, 3 and 4 appeared incorrectly.

The order of Figs. 2, 3 and 4 remains the same, and the correct legends are provided below:

Figure 2 Ultrasonography(B-scan) at the time of initial diagnosis showed thickening of the sclera and hyper-echoic banding in the temporal and inferior quadrants, consistent with retinal detachment (2**A**). Eight months after stopping treatment, the retina was completely reattached (2**B**)

Figure 3 OCT at the time of initial diagnosis revealed subretinal fluid in the macula OS(3**A**). One month later, OCT revealed significant resolution in the subretinal fluid (3**B**). Seven months after treatment, OCT showed minimal residual fluid in the fovea. (3**C)**

Figure 4 UBM at the time of initial diagnosis revealed a lesion with ciliary body granulomatous inflammation measuring 9.25*1.67*12.92 mm (4**A**). Seven months after treatment, UBM showed a significant reduction in the size of the ciliary body granuloma to approximately 7.82*0.75*2.54 mm (4**B**). Eight months after stopping treatment, UBM suggested that the size of the ciliary body granuloma was 1.96*0.23*1.16 mm (4**C**)

The original article has been updated.
